# Prefrontal-Dependent and Gender-Specific Modulation of Guilt Emotion on Human Early Visual Perception

**DOI:** 10.3390/bs15030333

**Published:** 2025-03-08

**Authors:** Mingyang Sun, Lihong Chen

**Affiliations:** 1 Research Center of Brain and Cognitive Neuroscience, Liaoning Normal University, Dalian 116029, China; 2 Key Laboratory of Brain and Cognitive Neuroscience, Liaoning Province, Dalian 116029, China

**Keywords:** guilt, Ebbinghaus illusion, gender difference, VLPFC, repetitive TMS

## Abstract

Negative emotions can shape human visual perception, which is mainly investigated using basic emotions such as fear. Whether guilt emotion, which is a negative moral emotion originating late in our evolutionary ancestry, has similar modulatory effects as basic emotions is largely unexplored. Here, we employed a dot estimation task to induce feelings of guilt and subsequently measured the Ebbinghaus illusion strength. The photos of victims’ faces were projected on the central circle of the Ebbinghaus configuration. The results showed that guilt significantly strengthened the illusion effect relative to control condition, which was observed only for female participants playing with same-gender partners and reversed to the opposite pattern with disruption of left ventrolateral prefrontal cortex. The findings suggest that guilt can sculpt early visual perception in a gender-specific and prefrontal-dependent manner, thus broaden our understanding of guilt emotion and have implications for relevant neuropsychiatric disorders.

## 1. Introduction

Emotions routinely influence how people see the world. Converging evidence shows that negative emotions shape basic aspects of visual perception. For instance, viewing fearful faces enhances contrast and orientation sensitivity (Bocanegra & Zeelenberg, 2009; Phelps et al., 2006). People tend to underestimate the time-to-collision for threatening (i.e., snakes and spiders) relative to neutral (i.e., butterflies and rabbits) stimuli (Vagnoni et al., 2012). Threatening primes (snakes and spiders) decrease the magnitude of visual size illusion (Hu et al., 2023). Likewise, people overestimate the size of threatening stimuli, such as spiders and an object on a collision course with their heads (Chen et al., 2016; Leibovich et al., 2016; Shiban et al., 2016; Vasey et al., 2012). Even individual differences in fear are linked to perceptual distortions, with higher claustrophobic fear corresponding to larger perceived size of near space (Lourenco et al., 2011). The above evidence suggests that negative emotions can enhance perceptual sensitivity in both time and space.

Guilt is a type of negative emotion that arises when an individual’s behavior violates internalized moral standards (Amodio et al., 2007). The feelings of guilt have been found to affect high-level cognitive abilities, such as impairing working memory performance (Cavalera & Pepe, 2014) and avoiding eye contact with the victims (Yu et al., 2017). Guilt also affects social attention, as demonstrated by enhanced facing-the-viewer bias and reduced gaze-cueing effect (Shen et al., 2018; Zhao et al., 2023). It remains unclear whether guilt impacts early perceptual processing in the same manner as basic emotions like fear. Guilt emotion is a relatively recent adaptation, whereas fear emotion originates early in our evolutionary ancestry (Gilead et al., 2016). Here, we investigated the influence of guilt emotion on visual size perception by using the classic Ebbinghaus illusion. The feelings of guilt were established and associated with a specific face (i.e., the victim) via a modified dot estimation task. The specific face was subsequently projected on the central circle of the Ebbinghaus configuration. Guilt might fundamentally alter the perceptual representation of the victim’s face, manifesting as changes in visual size perception. As guilt induces social avoidance of the victim (Yu et al., 2017), it is reasonable that the victim’s face displayed at the center of the Ebbinghaus configuration would attracts less attention, which would bring more uncertainty when estimating the size of the central circle, thus resulting in larger illusion effect. Moreover, previous studies have found that women are more susceptible to guilt emotion than men (Benetti-McQuoid & Bursik, 2005; Else-Quest et al., 2012; Ward & King, 2018), thereby we expected that the guilt-related modulation effect on visual size perception would be more pronounced for women than men.

Affective modulation of visual perception likely relies on prefrontal regulation of visual areas. A negative emotional state is associated with an increased connectivity from left prefrontal cortex to parietal and visual regions (Wyczesany et al., 2015). Greater correspondence between prefrontal cortex and primary visual cortex has been observed during the perception of negative pictures (Park et al., 2021). Notzon and colleagues found that inhibitory right dorsolateral prefrontal stimulation led to sustained increased processing of fearful faces relative to neutral faces in the right occipital region, and excitatory prefrontal stimulation resulted in the reverse pattern (Notzon et al., 2018). It remains to be determined whether guilt and basic negative emotions have common or distinct mechanisms when modulating early perceptual processing. While both basic and social emotions activate shared regions like the amygdala and inferior frontal gyrus, social emotions uniquely recruit the orbital and medial prefrontal cortex, and the superior temporal sulcus (Moll et al., 2002). The ventrolateral prefrontal cortices (VLPFC) are especially implicated in regulating social emotions (Li et al., 2022) like guilt (Morey et al., 2012; Zhu et al., 2019). To investigate the causal contribution of VLPFC to the guilt-related modulation of visual size perception, we disrupted the activity of left VLPFC via repetitive transcranial magnetic stimulation (rTMS). If this region played a causal role in this process, the guilt-related modulation effect would be greatly reduced or even vanish with the disruption of left VLPFC.

## 2. Methods

### 2.1. Participants

A total of 76 participants (20 males; mean age = 21.2 years, SD = 1.9 years) took part in the study, with 20 for Experiment 1 (10 males), 20 for Experiment 2 (10 males), and another 36 for Experiment 3 (all females). Sample size was determined based on power analyses following a previous relevant study (Hu et al., 2023). All participants had normal or corrected-to-normal eyesight and provided informed consent. They were naive to the purpose of the experiments. This study was approved by the institutional review board of Liaoning Normal University, and adhered to the tenets of the Declaration of Helsinki.

### 2.2. Stimuli

Stimulus presentation was controlled using MATLAB (R2020b; Mathworks, Natick, MA, USA) software with Psychtoolbox extensions (Brainard, 1997; Pelli, 1997). Photos of faces with a neutral expression (one male and one female) were used to represent simulated players. The Ebbinghaus illusion configuration consisted of a target circle (diameter = 1.1°) surrounded by four large or small circles (diameter = 1.7° and 0.6°, respectively). The photo of the ostensible player was projected on the target circle. On each trial, the initial diameter of a comparison circle varied ranging from 0.86° to 1.37° in 0.06° steps. Participants were positioned 57 cm from a gray computer screen (1920 × 1080 at 60 Hz), and their head was stabilized with a chin rest.

### 2.3. Procedure

At the beginning of the experiments, participants were told that they had to perform two tasks, with the first dot estimation task performed together with another person (a partner, role-played by a student), and the second size-matching task conducted by himself/herself.

**Figure 1:**
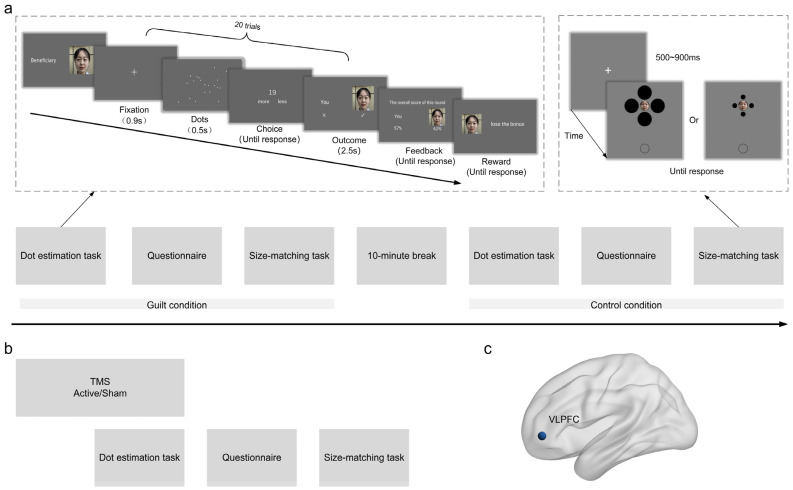
Experiment procedures and illustration of stimulation site. (**a**) In the dot estimation task, participants were required to perform a 20-round estimation of the number of dots together with a partner role-played by a student. In the size-matching task, the photo of the partner’s face was projected on the central circle of the Ebbinghaus configuration. The comparison circle was simultaneously presented below it. Participants were required to adjust the size of the comparison circle to match that of the central target with no time limit. (**b**) During the TMS experiment (Experiment 3), real or sham stimulation was applied 5 min before the start of the dot estimation task and persisted for another 5 min during the task. (**c**) Illustration of the stimulation site (i.e., left VLPFC) on the brain.

The procedures of Experiments 1 and 2 were the same, except that the participants and their partners were of the same gender in Experiment 1 and were of the opposite gender in Experiment 2. Participants performed the dot estimation task and the size-matching task twice (Figure 1a). In particular, they first completed the dot estimation task (e.g., the guilt condition), then performed the size-matching task. After a 10-min break, they completed another dot estimation task (e.g., the control condition), followed by the same size-matching task. The order of emotion condition was counterbalanced across participants.

The procedure of Experiment 3 was similar to that of Experiment 1, with the exception that 10-minute rTMS was applied before and during the dot estimation task (Figure 1b). Each participant conducted two sessions (active and sham TMS), the order of which was counterbalanced across participants with an interval of at least seven days. Half of the participants were in the guilt condition and the other half were in the control condition. On each session, participants received 5-minute stimulation over left VLPFC (Figure 1c) with no explicit task and then performed the dot estimation task and simultaneously received another 5-min stimulation. Immediately, in the following period, they conducted the size-matching task.

### 2.4. Dot Estimation Task

Participants were told that if both they and their partners achieved an accuracy rate of over 60%, one of them would receive a bonus. However, if either the participants or their partners had an accuracy of below 60%, neither of them would receive the bonus.

At the beginning of the dot estimation task, either the participant or the partner indicated by a face photo was designated as the bonus recipient. A fixation cross was presented for 0.9 s, followed by 20 white dots displayed for 0.5 s with their positions randomly generated. Then, one number (19 or 20) and two words, “more” and “less”, were presented on the screen. The participant had to determine whether the dots were more or less than the number as fast as possible. After one round of 20 repetitions, the accuracy for both the participant and the partner was shown for 2.5 s, which was predetermined by the experimenter. Finally, feedback on whether the participant or the partner earned or lost the bonus was provided on the screen.

Guilt emotion was manipulated by varying the outcome feedback to the participants. Each participant played two rounds with the partner under both the guilt and the control conditions. The bonus recipient was the participant in the first round, and the simulated partner in the second round. In the control condition, both the participant and the partner earned the bonus due to their good performance in the two rounds. In the guilt condition, the participant earned the bonus in the first round, whereas the partner lost the bonus due to the participant’s poor performance in the second round.

To fully assess the changes of emotional states induced by experimental manipulation, participants were asked to report their feelings based on their performance (i.e., sadness, shame, happiness, guilt, anger, and pride), using a 7-point scale (1 = not at all to 7 = very strongly), according to previous research (Zhu et al., 2017). The assessment took about one minute to complete.

### 2.5. Size-Matching Task

The Ebbinghaus configuration was presented at the screen center, and the comparison circle was simultaneously presented below it (8.6° from the screen center). Participants were required to adjust the size of the comparison circle to match that of the central target without a time limit. There were 11 trials for each condition (size of inducers: large or small).

### 2.6. Repetitive Transcranial Magnetic Stimulation (rTMS)

A PowerMAG TMS system (Mag and More, Berlin, Germany) in combination with a figure of 8-shaped coil (Double coil PMD70-pCool) was used for pulse delivery. TMS Navigation (Visor 2, ANT-Neuro, Berlin, Germany) was employed to determine stimulation site, to guide the placement and orientation of the coil, and to allow online tracking for minimizing deviations from the optimal site of stimulation. The coil was held tangentially to the skull and was oriented such that the coil-center was overlaying the site of left VLPFC. Individual resting motor threshold (RMT) was determined for the left abductor pollicis brevis muscle and defined as the minimum stimulus intensity that produced a motor evoked potential in at least 5 out of 10 trials. Stimulation intensity was set at 90% of the individual RMT. Participants received a 10-min 1-Hz train of pulses, which can produce inhibition of the stimulated site (Hilgetag et al., 2001; Mevorach et al., 2006).

Individual anatomical T1 weighted MRI scans were acquired using a 3T MR scanner (MR-750, GE medical systems, Milwaukee, WI, USA) and a magnetization-prepared rapid gradient echo sequence (echo time = 2.9 ms, repetition time = 6.7 ms, field of view = 256 mm, matrix = 256 × 256, flip angle = 8°, spatial resolution = 1 × 1 × 1 mm^3^). MNI coordinates of the stimulation site (left VLPFC: −50, 46, −2) were obtained from a relevant fMRI study (Morey et al., 2012). The coordinates were first projected on each individual reconstructed 3D anatomical MRI scan, which was then co-registered with the participant’s head to allow for precise positioning and online guiding of the coil.

### 2.7. Data Analysis

The illusion magnitude was calculated as the difference in the perceived size of the central target surrounded by small and large inducers relative to its physical size (%). Paired sample *t*-tests (two tailed) were adopted to compare the difference in the illusion magnitude between the control and the guilt conditions (Experiments 1 and 2) and between the active and sham stimulations (Experiment 3).

## 3. Results

### 3.1. Experiment 1

For the dot estimation task (Table 1), relative to control condition, participants reported higher levels of guilt in the guilt condition (mean difference = 2.80, *t*(19) = 5.22, *p* < 0.001, *d* = 1.17), indicating that the experimental manipulation was successful. Notably, in the guilt condition, the ratings of guilt were significantly higher than the ratings of other emotions (*p*s < 0.001), and females exhibited more guilt than males (*t*(18) = 4.03, *p* < 0.001, *d* = 1.80).

**Figure 2:**
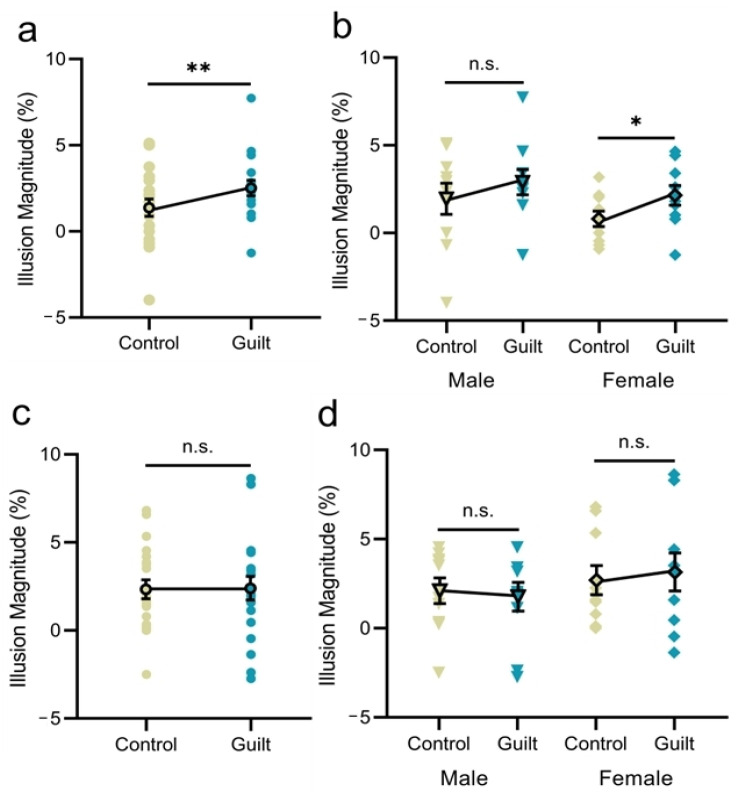
Results of Experiments 1 and 2. Illusion magnitude as a function of emotion condition when all participants were analyzed together or separately for males and females for Experiments 1 (**a**,**b**) and 2 (**c**,**d**). Error bars represent one standard error of the mean. Asterisk (*) indicates significance level of * *p* < 0.05, ** *p* < 0.01, and n.s. indicates non-significant *p*-value (*p* > 0.05).

For the size-matching task, the illusion magnitude for the guilt condition was significantly larger than the control condition (*t*(19) = 2.88, *p* = 0.010, *d* = 0.64; Figure 2a). When female and male participants were analyzed separately (Figure 2b), significant guilt-related modulation effect was observed for the females (*t*(9) = 2.38, *p* = 0.041, *d* = 0.75), rather than for the males (*t*(9) = 1.62, *p* = 0.140, *d* = 0.51).

### 3.2. Experiment 2

For the dot estimation task (Table 1), the ratings of guilt were significantly higher in the guilt condition than the control condition (mean difference = 2.65, *t*(19) = 8.55, *p* < 0.001, *d* = 1.91). Moreover, in the guilt condition, the ratings of guilt were significantly higher than that of other emotions (*p*s < 0.001).

For the size-matching task, there was no significant difference in the illusion magnitude between the guilt and the control conditions (*t*(19) = 0.14, *p* = 0.890, *d* = 0.03; Figure 2c). A similar pattern of results was observed when male and female participants were analyzed separately (males: *t*(9) = 0.78, *p* = 0.457, *d* = 0.18; females: *t*(9) = 0.66, *p* = 0.523, *d* = 0.21; Figure 2d).

### 3.3. Experiment 3

As significant guilt-related modulation effect was observed only for female participants playing with same-gender partners (Experiment 1) rather than with opposite-gender partners (Experiment 2), we recruited only female participants playing with same-gender partners in Experiment 3.

**Figure 3:**
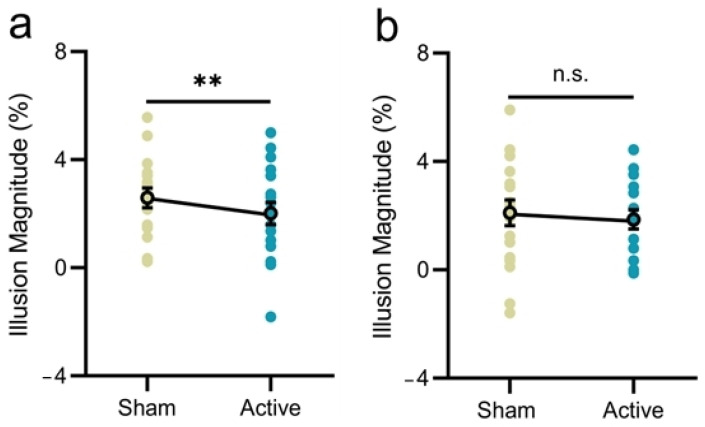
Results of Experiment 3. Illusion magnitude as a function of stimulation type in the guilt (**a**) and the control (**b**) conditions. Error bars represent one standard error of the mean. Asterisk (*) indicates significance level of ** *p* < 0.01, and n.s. indicates non-significant *p*-value (*p* > 0.05).

In the guilt condition (Table 1), the ratings of guilt were significantly higher than that of other emotions with sham stimulation (*p*s < 0.001), confirming the results of Experiments 1 and 2. Notably, in comparison with sham stimulation, inhibitory stimulation of left VLPFC significantly reduced the ratings of guilt (*t*(17) = −2.69, *p* = 0.015, *d* = 0.63), and significantly reduced the illusion magnitude (*t*(17) = −2.90, *p* = 0.010, *d* = 0.68; Figure 3a). However, in the control condition (Figure 3b), significant stimulation effect was not observed for neither the guilt emotion (*t*(17) = 1.29, *p* = 0.215, *d* = 0.30) nor the illusion effect (*t*(17) = −0.60, *p* = 0.556, *d* = 0.14).

## 4. Discussion

The present study aims to investigate whether guilt influences visual size perception and the causal role of left VLPFC in mediating this effect. Results showed that guilt significantly increased the Ebbinghaus illusion effect, and this was more pronounced for female participants played with same-gender partners (Experiment 1). However, this modulation effect disappeared with opposite-gender partners (Experiment 2) and reversed when the activity of the left VLPFC was disrupted by inhibitory rTMS (Experiment 3).

Previous studies that investigate affective modulation of visual size perception largely adopt basic emotions like fear and anger. For instance, people are apt to overestimate the size of fearful stimuli, and that of neutral stimuli when in a fearful state (Chen et al., 2016; Leibovich et al., 2016; Lourenco et al., 2011; Shiban et al., 2016; Vasey et al., 2012). A threatening prime can decrease subsequent size illusion strength relative to a neutral prime (Hu et al., 2023). Similarly, when negative pictures were projected on the central target of the Ebbinghaus configuration, the illusion effect was greatly reduced (Van Ulzen et al., 2008). However, to our knowledge, no study has as yet explored the modulation of more complex social and moral emotions on visual size perception. The present study adopted the dot estimation task to induce the feelings of guilt and found the opposite patterns of results in comparison with fear emotion, i.e., guilt emotion significantly increased the size illusion strength relative to control condition. The above findings suggest that basic and social emotions may exert distinct influences on early visual perception.

In contrast to basic emotions, social emotions require insight into the mental states of other people. Guilt can trigger social avoidance (Yu et al., 2017), i.e., individuals experiencing guilt tend to avoid attending to the faces of victims. In the current study, the face of the partner (i.e., the victim) was displayed on the central circle of the Ebbinghaus configuration. It is conceivable that participants in the guilt condition would avoid looking at the faces of the victims, thereby allocating less attention to the central target, which increased the uncertainty of the size estimation of the central target, thus further leading to the enhancement of the size illusion magnitude. In Hu et al.’s study, a threatening (snakes or spiders) or neutral (rabbits or horses) animal picture was displayed before the presentation of the Ebbinghaus configuration. The threatening prime might attract more attention and thus leave less attention for the subsequent processing of surround inducers, resulting in a reduced illusion effect. Taken together, though the modulation of both guilt and fear emotions on visual size perception might take effect via attention, they probably rely on distinct functions of attention, i.e., attentional avoidance and attraction, respectively.

Consistent with prior work showing that females are more prone to guilt than males, the present study revealed a gender difference not only in guilt itself but also in its perceptual consequences. Specifically, the guilt-related enhancement of visual size illusion was observed only for female and not male participants. The possible reason for this discrepancy could be that males are better at regulating negative emotions (Birditt & Fingerman, 2003; Cai et al., 2016), whereas females are generally considered to be more emotional and guilt prone, and are more susceptible to the impact of negative events in life and mood disorders (Cohen et al., 2012; Sloan & Kornstein, 2003; Yuan et al., 2009). Furthermore, females are inherently more inclined to establish and emphasize interpersonal relationships (Cross & Madson, 1997; Kashima et al., 1995; Lewis, 1985), whereas males display stronger self-relevant biases during the process of socialization (Cross & Madson, 1997; Russell et al., 2017). Therefore, it is plausible that women’s greater propensity to experience guilt, relative to men, underlies the more pronounced impact of guilt on early visual perceptual processes observed in female participants.

Intriguingly, this gender difference in guilt-related modulation was only observed with same-gender partners, and it vanished with opposite-gender partners. Same-gender friendships of females are characterized by intimacy and cooperation (Keener et al., 2012), and female adolescents express more empathetic sadness towards same-gender individuals than opposite-gender ones (Stuijfzand et al., 2016). Therefore, we speculated that the more pronounced guilt-related modulation effect for same-gender partners could be due to the fact that females are more susceptible to the guilt emotion associated with same-gender partners. This conjecture was confirmed by direct comparison of the ratings of guilt between same- and opposite-gender partners, with the former being significantly higher than the latter (*t*(18) = 2.56, *p* = 0.020, *d* = 1.14).

The present study further showed that, when the activity of the left VLPFC was temporally disrupted by rTMS, the guilt-related enhancement of visual size illusion was significantly reduced and even reversed to the opposite pattern. The VLPFC is especially associated with negative emotions (Achterberg et al., 2018; Chick et al., 2020; Hsu et al., 2020; Miller et al., 2019). Abnormal activation of the VLPFC is observed in a wide range of emotional disorders, including anxiety disorder (Yokoyama et al., 2015), major depressive disorder (Henderson et al., 2014), bipolar disorder (Phillips & Swartz, 2014), and post-traumatic stress disorder (Keding & Herringa, 2016). The emotion-type hypothesis proposes that the basic and nonsocial emotions are modulated by the right hemisphere, and social emotions are modulated by the left hemisphere (Ross, 2021). In support of this theory, the left VLPFC has been found to be involved in the experience of guilt emotion (Morey et al., 2012; Zhu et al., 2019). The current findings suggest that left VLPFC not only involves in the processing of guilt emotion, but is also associated with the guilt-related modulation of early visual perception. The processing of visual size illusions involves occipital and parietal cortex (Chen et al., 2024; Wang et al., 2021; Wu et al., 2023), and converging evidence has found direct connections projecting from prefrontal cortex to occipital and parietal regions (Bressler et al., 2008; Ester et al., 2015; Park et al., 2021; Ruff et al., 2008; Silver & Kastner, 2009; Wyczesany et al., 2015). Thus, it seems likely that the guilt-related modulation effect on visual size perception might rely on the feedback projections from VLPFC to occipital and parietal cortex. The exact neural networks underlying this effect remain to be clarified.

In summary, the present study reveals that guilt emotion can affect early visual perception in a gender-specific and prefrontal-dependent manner. The findings provide clear evidence that social emotions not only affect social cognition, but also take effect on early visual perception, thus shedding new light on our understanding of the interaction of emotion and cognition, and having implications for neuropsychiatric disorders that involve social emotions. Future research could employ alternative inductions and measures to rule out the influence of other co-occurring emotions and better isolate the specific effects of guilt.

## Figures and Tables

**Table 1:** Ratings of emotional states in the dot estimation task (M ± SD).

Emotion	Experiment1		Experiment2		Experiment3			
	Guilt	Control	Guilt	Control	Sham		Active	
					Guilt	Control	Guilt	Control
Guilt	5.05 ± 2.01	2.25 ± 1.55	5.30 ± 1.13	2.65 ± 1.23	5.33 ± 1.94	1.28 ± 0.58	4.11 ± 2.03	1.50 ± 0.62
Shame	3.95 ± 2.24	2.15 ± 1.35	3.95 ± 1.93	2.20 ± 1.06	3.50 ± 2.18	1.28 ± 0.58	3.17 ± 2.15	1.56 ± 0.71
Anger	1.60 ± 1.14	1.50 ± 0.83	2.45 ± 1.47	1.50 ± 0.89	2.17 ± 1.54	1.22 ± 0.43	1.56 ± 1.34	1.39 ± 0.61
Pride	2.60 ± 1.88	3.75 ± 1.94	2.25 ± 1.21	4.25 ± 1.25	2.78 ± 1.40	3.28 ± 2.14	3.06 ± 1.47	3.61 ± 2.00
Sadness	3.40 ± 1.60	1.80 ± 1.28	3.50 ± 1.67	1.65 ± 0.93	3.00 ± 1.88	1.22 ± 0.55	2.61 ± 1.85	1.33 ± 0.59
Happiness	3.00 ± 1.81	4.55 ± 1.82	2.80 ± 1.51	5.25 ± 1.41	2.94 ± 1.39	4.11 ± 1.88	4.11 ± 1.18	4.44 ± 1.85

## Data Availability

None of the experiments reported in this article were preregistered. All data have been made publicly available via OSF and can be accessed at https://osf.io/p65ks/ (accessed on 5 September 2024).
